# Differential Estrogenic Actions of Endocrine-Disrupting Chemicals Bisphenol A, Bisphenol AF, and Zearalenone through Estrogen Receptor α and β *in Vitro*

**DOI:** 10.1289/ehp.1104689

**Published:** 2012-04-11

**Authors:** Yin Li, Katherine A. Burns, Yukitomo Arao, Colin J. Luh, Kenneth S. Korach

**Affiliations:** Receptor Biology Section, Laboratory of Reproductive and Developmental Toxicology, National Institute of Environmental Health Sciences, National Institutes of Health, Department of Health and Human Services, Research Triangle Park, North Carolina, USA

**Keywords:** BPA, BPAF, ERα, ERα mutants, ERβ, ERE, estrogen, zearalenone

## Abstract

Background: Endocrine-disrupting chemicals (EDCs) are widely found in the environment. Estrogen-like activity is attributed to EDCs, such as bisphenol A (BPA), bisphenol AF (BPAF), and zearalenone (Zea), but mechanisms of action and diversity of effects are poorly understood.

Objectives: We used *in vitro* models to evaluate the mechanistic actions of BPA, BPAF, and Zea on estrogen receptor (ER) α and ERβ.

Methods: We used three human cell lines (Ishikawa, HeLa, and HepG2) representing three cell types to evaluate the estrogen promoter activity of BPA, BPAF, and Zea on ERα and ERβ. Ishikawa/ERα stable cells were used to determine changes in estrogen response element (ERE)-mediated target gene expression or rapid action-mediated effects.

Results: The three EDCs showed strong estrogenic activity as agonists for ERα in a dose-dependent manner. At lower concentrations, BPA acted as an antagonist for ERα in Ishikawa cells and BPAF acted as an antagonist for ERβ in HeLa cells, whereas Zea was only a partial antagonist for ERα. ERE-mediated activation by BPA and BPAF was via the AF-2 function of ERα, but Zea activated via both the AF-1 and AF-2 functions. Endogenous ERα target genes and rapid signaling via the p44/42 MAPK pathway were activated by BPA, BPAF, and Zea.

Conclusion: BPA and BPAF can function as EDCs by acting as cell type–specific agonists (≥ 10 nM) or antagonists (≤ 10 nM) for ERα and ERβ. Zea had strong estrogenic activity and activated both the AF-1 and AF-2 functions of ERα. In addition, all three compounds induced the rapid action-mediated response for ERα.

Endocrine-disrupting chemicals (EDCs) alter the function of the endocrine system and consequently cause adverse health effects (Colborn et al. 1993; Diamanti-Kandarakis et al. 2009). In the 1970s, researchers introduced the concept of endocrine disruption regarding the hazards caused by xenobiotic exposure to wildlife and humans. Animal studies are typically performed using relatively high acute doses of EDCs; however, the mechanistic effects of low-dose human exposure to EDCs are unknown. EDCs encompass a variety of chemical classes, including hormones, plant constituents, pesticides, compounds used in the plastic industry, and other industrial by-products and pollutants (Colborn et al. 1993). Many are pervasive, are widely dispersed in the environment, and can be found at higher concentrations in wildlife due to bioaccumulation (Boehme et al. 2009; Colborn et al. 1993).

Bisphenol A (BPA) is a chemical used primarily in the manufacture of polycarbonate plastics and as a nonpolymer additive to other plastic (Wetherill et al. 2007). BPA measured in adult and fetal serum ranges from 0.5 to 40 nM, with exposure coming from food, beverages, and the environment (Welshons et al. 2006). Bisphenol AF (BPAF) is a fluorinated derivative of BPA used in polycarbonate copolymers in high-temperature composites, electronic materials, gas-permeable membranes, and specialty polymer applications. Humans and wildlife populations are exposed to levels of BPA/BPAF that have been reported to cause adverse reproductive and developmental effects in several different *in vitro* and *in vivo* models (Akahori et al. 2008; Bay et al. 2004; Bermudez et al. 2010). Zearalenone (Zea), also known as F-2 toxin, is a nonsteroidal estrogenic mycotoxin produced by various species of *Fusarium*. It is heat-stable and is found worldwide in cereal crops and in bread made from maize, barley, oats, wheat, rice, and/or sorghum (Tanaka et al. 1988). Analogs of Zea constitute an important class of EDCs that have estrogen receptor (ER) activity. Zea can cause physiological alterations of the reproductive tract by disturbing ovulation and affecting conception, implantation, and fetal development (Parveen et al. 2009). However, the mechanism of action of BPA, BPAF, and Zea involving ERα and ERβ is not well understood in regard to dose dependence, functionality, tissue selectivity, and rapid action responses. Chemical structures of BPA, BPAF, and Zea are shown in Supplemental Material, Figure 1 (http://dx.doi.org/10.1289/ehp.1104689).

**Figure 1:**
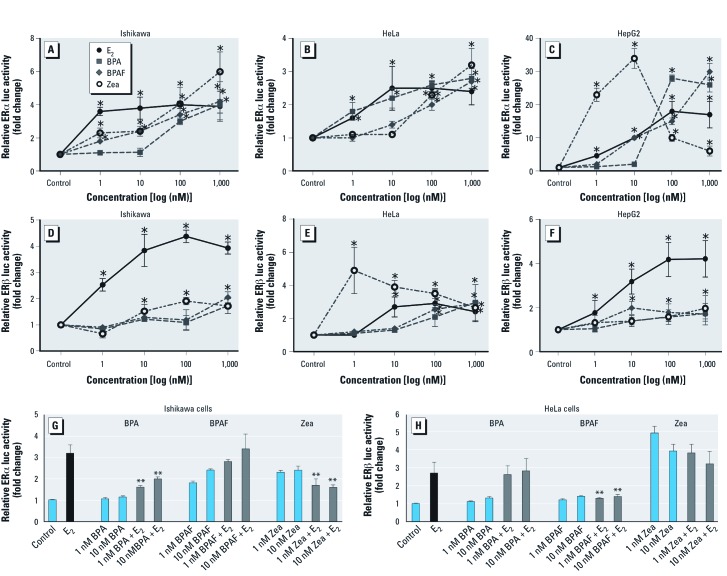
BPA, BPAF, and Zea act as agonists or antagonists for ERα and ERβ. (*A–F*) Dose–response curves for ERα (*A–C*) or ERβ (*D–F*) in Ishikawa (*A,D*), HeLa (*B,E*), and HepG2 (*C,F*) cells transfected with ERE-luc, pRL‑TK, and either pcDNA/WT‑ERα or pcDNA/WT‑ERβ plasmids and treated with vehicle (control), 1, 10, 100, or 1,000 nM E_2_, BPA, BPAF, or Zea for 18 hr; ERE-mediated ERα and ERβ activation was detected by luciferase reporter assays. Data shown are mean ± SEM fold change relative to control for three independent experiments. (*G,H*) BPA, BPAF, and Zea antagonize E_2_-mediated ERα activation. Cells transfected with ERE-luc, pRL‑TK, and pcDNA/WT ERα (Ishikawa cells) or pcDNA/WT ERβ (HeLa cells) plasmids were treated with vehicle (control), 10 nM E_2_, or BPA, BPAF, or Zea (1 or 10 nM alone or with E_2_ for 18 hr, and ERE-mediated ERα and ERβ activation was detected by luciferase reporter assay. Data shown are mean ± SEM fold change relative to control for three independent experiments. *p < 0.05 compared with control. **p < 0.05 compared with 10 nM E_2_ treatment.

ERα and ERβ belong to the nuclear receptor superfamily of ligand-inducible transcription factors. Several biological effects of estrogen (17β-estradiol; E_2_) and E_2_-like compounds and ligands are mediated through the ERs. There are three main mechanisms of action for the ERs: *a*) the classical nuclear, genomic mechanism, in which ligand-bound ERs interact directly with estrogen response elements (EREs) to regulate transcription; *b*) the nonclassical nuclear, genomic “tethered” mechanism, in which ER interacts with other transcription factors on sites such as AP-1 or Sp1 to regulate gene expression; and *c*) the rapid nongenomic action mechanism, which activates signaling cascades involving p44/42 MAPK (p44/42 mitogen-activated protein kinase), s*rc*, and Akt (Burns et al. 2011; Hall and McDonnell 2005).

ERα and ERβ, like other nuclear receptors, have A/B, C, D, and E/F domains. Each domain can act independently, but for full functionality, proper spatial orientation is necessary for transactivation of target genes (Kumar and Thompson 1999; Zwart et al. 2010). In addition, two acidic activation domains mediate the ligand-dependent transcriptional activity of ERα: activation function-1 (AF-1) in the A/B domain, and AF-2, a hormone-dependent function, in the E/F domain (Hall and McDonnell 2005). Several ERα mutants have been used in *in vitro* or *in vivo* models to evaluate the mechanistic signaling action of E_2_ on the function of ERα. Our laboratory has recently demonstrated that the H1 mutation in the hinge region disrupts nuclear localization and prevents tethered-mediated responses, but retains ERE-mediated genomic action (Burns et al. 2011). The DNA binding domain (DBD) mutation (also known as AA ERα) prevents direct DNA binding but maintains tethered-mediated gene activities (Jakacka et al. 2002; O’Brien et al. 2006). The E1 mutation in the A/B domain disrupts AF-1 function but not AF-2 function (Couse et al. 1995). The AF-2 mutation in the E/F domain disrupts AF-2 but can be activated via the AF-1 function by the estrogen antagonist ICI 182,780 (ICI) (Arao et al. 2011; Kumar and Thompson 1999; Mader et al. 1993; Mahfoudi et al. 1995).

In the present study, we used three different human cell lines to evaluate dose- and cell-specific estrogenic or antagonistic ERE-mediated responses for ERα or ERβ to BPA, BPAF, and Zea. The biological function of ERα was analyzed using wild-type (WT) ERα and specific ERα mutants (H1, AA, E1, and AF-2) after treatment with BPA, BPAF, and Zea. We also explored the effects of these EDCs on estrogen-mediated target genes, and on rapid nongenomic effects through p44/42 MAPK and the *src* family of tyrosine kinase signaling pathways.

## Materials and Methods

*Reagents and antibodies.* We purchased E_2_ from Sigma-Aldrich (St. Louis, MO), ICI from Tocris Bioscience (Ellisville, MO), and BPA (CAS no. 80-05-7), BPAF (CAS no. 1478-61-1), and Zea (CAS no. 17924092-4) from Midwest Research Institute (Kansas City, MO). The p44/42 MAPK inhibitor PD 98059 and *src* family tyrosine kinase inhibitor PP2 were purchased from Calbiochem (San Diego, CA). We obtained anti-ERα antibody (sc-542) from Santa Cruz Biotechnology (San Diego, CA), anti-β-actin antibody (A2228) from Sigma-Aldrich, Phosphorylated (phospho)-p44/42 MAPK antibody (9101), total p44/42 MAPK antibody (9102), phospho-GSK-3β antibody (9323), total GSK-3β antibody (9315), phospho-Akt antibody (4060), and total Akt antibody (9272) from Cell Signaling Technology (Danvers, MA).

*Plasmids.* pGL3/3xERE luciferase reporter (ERE-Luc), pcDNA/mouse (m)WT ERα, and pcDNA/mE1 mutant were described previously by Mueller et al. (2003); the pcDNA/mH1, pcDNA/mAA, and pcDNA/mAF-2 mutants have also been described previously (Burns et al. 2011; Jakacka et al. 2002; Winuthayanon et al. 2009). The pcDNA/SRC2 plasmid was a gift from D. McDonnell, and pCMV/p300 from S. Kato.

*Cell lines and tissue culture.* HeLa human cervical epithelial cancer cells and HepG2 human hepatocellular cancer cells were purchased from ATCC (Manassas, VA). Ishikawa human endometrial adenocarcinoma cells and the stable cell lines Ishikawa/vector (Ishikawa/vec) and Ishikawa/WT ERα (Ishikawa/ERα) have been described previously (Burns et al. 2011; Mueller et al. 2003). HeLa cells were maintained in phenol-red free Dulbecco’s Modified Eagle Medium (DMEM) supplemented with 10% fetal bovine serum (FBS; BenchMark; Gemini Bio-Products, West Sacramento, CA). The HepG2 and Ishikawa cell lines were maintained in phenol-red free DMEM:F12 medium supplemented with 10% FBS. The stable cell lines, Ishikawa/vec and Ishikawa/ERα, were maintained in phenol-red free DMEM:F12 supplemented with 10% FBS and geneticin (G418; 1.4 mg/mL). For serum-starved conditions, 10% stripped FBS (sFBS; Thermo Scientific, Waltham, MA) was substituted for FBS (starve medium).

*Transient transfection and luciferase assay.* Cells were seeded in 24-well plates and incubated in serum-starved medium overnight. A total of 0.5 μg DNA, including 0.2 μg of expression plasmid, 0.2 μg of reporter plasmid, and 0.1 μg of pRL-TK plasmid, were transfected overnight using Effectene Transfection Reagent (QIAGEN, Valencia, CA) according to the manufacturer’s protocol. Eight hours after changing to fresh starve medium, cells were treated with EDCs (1, 10, 100, or 1,000 nM) or vehicle (0.01% DMSO). For experiments with SRC2 or p300, cells were transfected with 0.8 μg of reporter plasmids, including 0.1 μg ERα, 0.4 μg coactivator, and 0.1 μg pRL-TK plasmids. Luciferase assays were performed using the Dual-Luciferase Reporter Activity System (Promega, Madison, WI). Transfection efficiency was normalized by renilla luciferase using pRL-TK plasmid. Fold changes were calculated relative to the vehicle. Data presented are multiple replicates from three independent experiments.

*RNA extraction and real-time polymerase chain reaction (PCR).* Total RNA was extracted using the QIAGEN RNeasy Mini Kit (QIAGEN). We performed first-strand cDNA synthesis using Superscript Reverse Transcriptase (Invitrogen). mRNA levels of ER target genes were measured using SYBR green assays (Applied Biosystems, Foster City, CA). Cycle time (Ct) values were obtained using the ABI PRISM 7900 Sequence Detection System and analysis software (Applied Biosystems). Each sample was quantified against its β-actin transcript content. Experiments were repeated three times, and results are presented as fold change ± SD. The sequences of primers used in real-time PCR are shown in Supplemental Material, Table 1 (http://dx.doi.org/10.1289/ehp.1104689).

*Protein extraction and Western blot analysis.* We prepared whole cell lysates using the BD TransFactor Extraction Kit (BD Biosciences, Palo Alto, CA). β-Actin was used as a loading control. Western blot analysis has been described previously (Burns et al. 2011). Briefly, the samples were loaded on a SDS-PAGE gel, heated, and separated by electrophoresis. The proteins were then electrotransferred onto nitrocellulose membranes and blocked for 2 hr with phosphate-buffered saline (PBS) containing 5% nonfat milk. The blots were incubated with primary antibodies overnight at 4°C, rinsed with PBS plus Tween-20, and then incubated with the appropriate horseradish peroxidase–conjugated secondary antibodies at room temperature for 1 hr. Immunoreactive products were detected with the ECL (enhanced chemiluminescence) system (Amersham Pharmacia, Piscataway, NJ).

*MAPK analysis.* The methods used for MAPK analysis were described previously by Burns et al. (2011). Briefly, cells were seeded and cultured in phenol red–free medium with 10% sFBS for 2 days; medium was then replaced with serum-free medium. Cells were pretreated without or with 10 µM inhibitors for 1 hr, followed by the addition of 100 nM E_2_, 1,000 nM BPA, 1,000 nM BPAF, or 1,000 nM Zea for 10 min. Cells were placed on ice, washed with cold PBS, and lysed in ice-cold lysis buffer for 30 min, and sonicated for 15 sec on ice. The supernatant (2 μg) was used for Western blot analysis as described above.

*Statistical analysis.* We performed one-way analysis of variance (ANOVA) with Tukey’s post test and two-way ANOVA with Bonferroni post test using GraphPad Prism, version 5.00 (GraphPad Software Inc., La Jolla, CA).

## Results

*Estrogenic activation of ER*α *and ER*β *by BPA, BPAF, and Zea.* To evaluate ERE-mediated transcriptional activity of ERα and ERβ, we examined promoter activation in Ishikawa, HeLa, and HepG2 cells. The luciferase reporter assay system was used to determine differential treatment effects of BPA, BPAF, and Zea on ERα or ERβ in cells derived from different tissues. Reporter activation of ERE-luc in response to E_2_, BPA, BPAF, or Zea was observed only with both ERα and ERβ expression plasmids but not with pcDNA control plasmid.

For ERα activation in Ishikawa cells, E_2_ activated the ERE-luc reporter at a concentration of 1 nM (4-fold induction) (Figure 1A). At low concentrations (≤ 10 nM), BPAF and Zea had weak estrogenic activity compared with E_2_, but stronger activation was observed at higher concentrations (1,000 nM). In contrast, 100 nM BPA was required for activation in these cells. Compared with Ishikawa cells, HeLa cells were less active, with only a 2.5-fold increase in ERE-mediated ERα activation in response to E_2_ (10 nM) (Figure 1B). ERα activation in response to BPA resembled that for E_2_, whereas induction by BPAF and Zea did not occur except with higher concentrations (≥ 100 nM). HepG2 cells were highly responsive to E_2_, with 10- to 20-fold increases in ERE-mediated transactivation at 10–1,000 nM E_2_ (Figure 1C). Interestingly, BPA activity was not evident for concentrations ≤ 100 nM, whereas Zea showed strong activation at 1 and 10 nM but less activity at higher concentrations. All activations occurred in a concentration-dependent manner in the three cell lines tested, with the exception of reduced activity of ERα activation when Zea was used at higher concentrations (≥ 100 nM).

In Ishikawa and HepG2 cells, ERβ was weak in response to the three EDCs compared with E_2_ (Figure 1D,F). In HeLa cells, ERβ responses to 100 or 1,000 nM BPA or BPAF were similar to those of E_2_, whereas the ERβ response was stronger for 1 nM Zea than for E_2_ (Figure 1E). In addition, the pure ER antagonist, ICI, blocked ERα and ERβ transcriptional activity in response to all three EDCs [see Supplemental Material, Figure 2 (http://dx.doi.org/10.1289/ehp.1104689)]. Taken together, these results demonstrate that BPA, BPAF, and Zea can activate ERE-mediated transcription in different cell types via ERα and ERβ, and that the estrogenic activity of each compound is cell type and concentration dependent.

**Figure 2:**
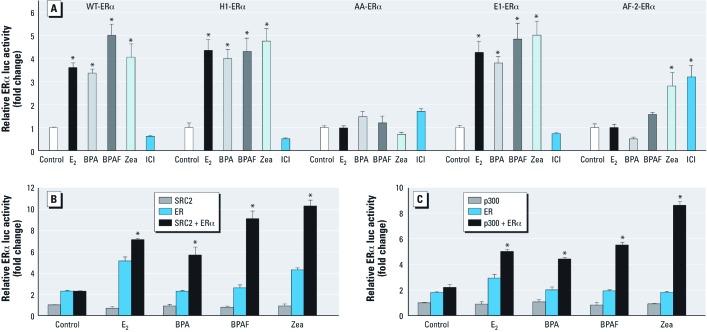
Functional analysis of BPA, BPAF, and Zea on WT and ERα mutants and coactivation of ERα by SRC2 or p300 in Ishikawa cells. (*A*) For functional analysis, cells transfected with ERE-luc, pRL‑TK, and pcDNA/WT ERα, pcDNA/H1 ERα, pcDNA/AA ERα, pcDNA/E1 ERα, or pcDNA/AF2 ERα plasmid were treated with vehicle, 10 nM E_2_, 100 nM BPA, 100 nM BPAF, 100 nM Zea, or 100 nM ICI for 18 hr, and ERα-ERE–mediated activity was detected by luciferase reporter assay. Data shown are mean ± SE fold change relative to control for three independent experiments relative to control. (*B,C*) Coactivation of ERα by SRC2 (*B*) or p300 (*C*) in cells transfected with ERE-luc, pRL‑TK, and pcDNA/SRC2 or p300, pcDNA/WT ERα, or pcDNA/SRC2 or p300 plus pcDNA/WT ERα plasmids and treated with the vehicle, 10 nM E_2_, or 100 nM BPA, BPAF, or Zea for 18 hr. ERE-mediated activation was detected by luciferase reporter assay. Data shown are mean ± SE fold change relative to control for three independent experiments relative to control. *p < 0.05 compared with control. **p < 0.05 compared with the vehicle for co-transfections.

*Activity of ER*α *and ER*β *is antagonized by low doses of BPA, BPAF, and Zea.* The EDCs showed weak activity in certain cell types at low doses. We therefore investigated antagonistic effects of BPA, BPAF, and Zea on ERα and ERβ using the ERE-mediated reporter assay system. Cells were transiently transfected with ERα or ERβ expression plasmids and then treated with 1 or 10 nM BPA, BPAF, or Zea with or without 10 nM E_2_ co-treatment. We observed antagonistic effects of BPA and Zea on ERα only in Ishikawa cells (Figure 1G). Both concentrations of BPA and Zea inhibited 70–80% of the E_2_-ERE–mediated reporter activity. At 1 and 10 nM, BPAF and Zea weakly activated ERα, but BPAF did not inhibit E_2_ activation. In addition, Zea (1 and 10 nM) induced ERα E_2_-ERE–mediated reporter activity, but E_2_-mediated activation was inhibited with E_2_ co-treatment (Figure 1G). We observed no antagonistic effects of these three EDCs on ERα in HeLa or HepG2 cells (data not shown).

For ERβ, E_2_-ERE–mediated reporter activity was reduced in HeLa cells treated with BPAF but not with BPA or Zea treatment (Figure 1B, 1H). None of the EDCs showed antagonistic effects on ERβ in Ishikawa or HepG2 cells (data not shown). Data demonstrate that low doses of BPA and BPAF (≤ 10 nM) antagonized ERα activity and ERβ activity, respectively; however, these effects were cell-type specific.

*Effects of BPA, BPAF, and Zea on ER*α *functionality.* To link BPA-, BPAF-, and Zea-mediated activation to a specific ERα functionality, we used four ERα mutants: H1-ERα (ERE-mediated activation, but no tethered-mediated activation), AA-ERα (tethered-mediated activation, but no ERE-mediated activation), E1-ERα (AF-1 inactive), and AF-2-ERα (AF-2 inactive). We observed estrogenic effects of BPA, BPAF, and Zea at 100 nM with WT-ERα in Ishikawa cells (Figure 1A). Therefore, we used this concentration in the mutant experiments. Cells were transiently transfected with an ERE-luc reporter plasmid and ERα mutant expression plasmids. Reporter activity was calculated for each mutant relative to activity in the vehicle control (Figure 2A). Responses to BPA, BPAF, and Zea were similar with H1-ERα and WT-ERα; however, none of the EDCs showed activation of AA-ERα; this is consistent with ERE-mediated activity, in which ERE reporter activity in response to BPA, BPAF, and Zea with E1-ERα (AF-1 inactive) was similar to activity with WT-ERα (Figure 2A). In addition, Zea activated the AF-2 mutant to an extent that was similar to ICI, which activates AF-2-ERα via the AF-1 function. Overall, these data indicate that ERE-mediated activation by BPA and BPAF was via the AF-2 function on ERα, whereas Zea activated ERα via both AF-1 and AF-2 functions.

In determining a role for ERα and activation with coactivators, we found that both SRC2 and p300 coactivated ERα-ERE–mediated activity with all three EDCs (Figure 2B,C). These data indicated that SRC2 and p300 act as ERα coactivators to increase transactivation in the presence of BPA, BPAF, and Zea, consistent with their AF-2 functionality.

**Figure 3:**
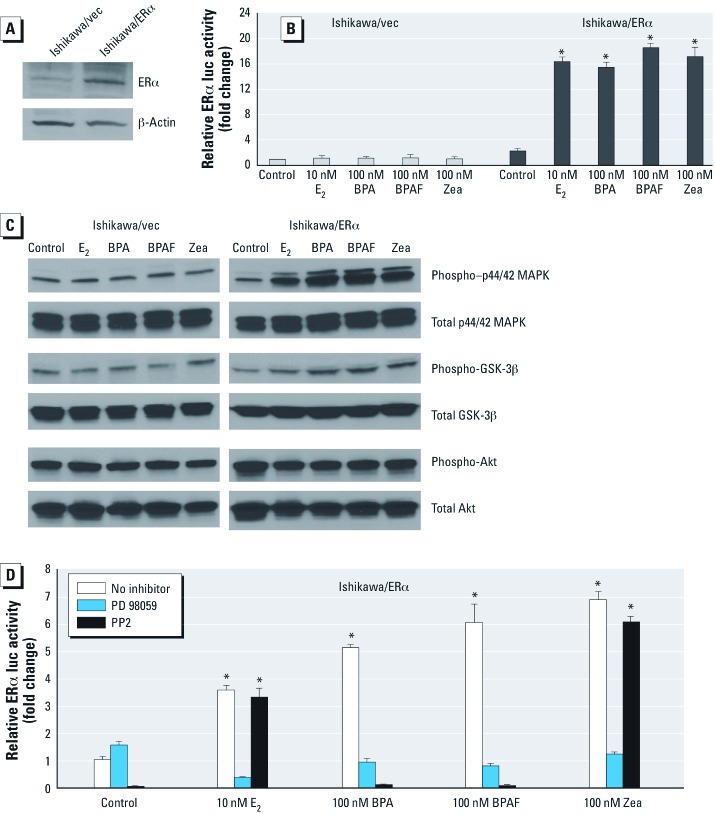
BPA, BPAF, and Zea affect p44/42 MAPK and *src *tyrosine kinase pathways in Ishikawa/ERα–stable cells. (*A*) Detection of ERα protein expression by Western blot in whole cell lysates prepared from Ishikawa/vec or Ishkawa/ERα cells. (*B*) ERE-mediated activity in cells transiently transfected with ERE-luc and pRL‑TK plasmids and treated with vehicle, 10 nM E_2_ or 100 nM BPA, BPAF, or Zea. Activity was detected by luciferase reporter assays, and data are mean ± SEM fold change relative to control for three independent experiments. (*C*) Western blot detection of phospho-p44/42 MAPK, phospho-GSK-3β, and phospho-Akt activation by 100 nM E_2_, 1,000 nM BPA, 1,000 nM BPAF, or 1,000 nM Zea. (*D*) Effect of PD 98059 and PP2 on the induction of *PR* gene expression by vehicle (control), 10 nM E_2_ or 100 nM BPA, BPAF, or Zea. *PR* transcripts were quantified by real time-PCR, and results are presented as mean ± SEM fold change relative to control for three independent experiments. *p < 0.05 compared with control.

*BPA and BPAF activate genes via p44/42 MAPK and* src *tyrosine kinase pathways, but Zea activates only through the p44/42 MAPK signaling pathway.* To examine phosphorylation events, we investigated the involvement of rapid action responses by BPA-, BPAF-, and Zea-mediated signaling pathways in Ishikawa cells stably expressing the vector control or WT-ERα (Burns et al. 2011). First, we confirmed ERα expression in Ishikawa/ERα cells by Western blot (Figure 3A) and then confirmed ERE-mediated activation by E_2_, BPA, BPAF, and Zea using the reporter assay system (Figure 3B). E_2_, BPA, BPAF, and Zea induced phospho-p44/42 MAPK in Ishikawa/ERα cells but not in Ishikawa/vec cells (Figure 3C), suggesting ERα-dependent and ligand-dependent activation. In addition, phospho-GSK-3β expression was only weakly induced by E_2_ and the EDCs, and we observed no increase in phospho-Akt in response to E_2_ or the EDCs in this model system (Figure 3C).

We next examined the effect of two specific kinase inhibitors—PD 98059 (MAPK inhibitor) and PP2 (*src* family tyrosine kinase inhibitor)—on BPA-, BPAF- and Zea-mediated expression of progesterone receptor (*PR*), a classic ER target gene, in Ishikawa/ERα cells. Both inhibitors blocked BPA- and BPAF-mediated endogenous *PR* gene expression, suggesting that BPA and BPAF are involved in the p44/42 MAPK and tyrosine kinase *src* pathways (Figure 3D). In contrast, induction of *PR* expression by E_2_ and Zea was inhibited by PD 98059 but not by PP2. Thus, while all three EDCs appeared to activate the p44/42 MAPK pathway in an ER-dependent manner, other kinase signaling pathways, such as the tyrosine kinase *src*, may also involve BPA and BPAF activation.

**Figure 4:**
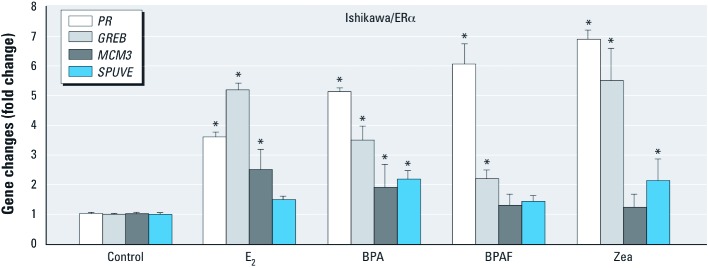
BPA, BPAF, and Zea regulate expression of the ER target genes *PR*, *GREB*, *MCM3*, and *SPUVE* in Ishikawa/ERα cells. Ishikawa/ERα cells were treated with vehicle (control), 10 nM E_2_, or 100 nM BPA, BPAF, or Zea for 18 hr and total RNA was extracted; mRNA levels of *PR*, *GREB*, *MCM3* and *SPUVE* were quantified by real-time PCR. Results are presented as fold change (mean ± SEM) relative to the control for three independent experiments. *p < 0.05 compared with control.

*BPA, BPAF, and Zea induced expression of ER target genes.* We confirmed ERα-dependent responses to BPA, BPAF, and Zea by detecting endogenous gene expression of *PR*, *GREB1* (gene regulation by estrogen in breast cancer 1), *MCM3* (minichromosome maintenance complex component 3), and *SPUVE* (also known as *PRSS23*; a member of the trypsin family of serine proteases) by real time-PCR (Figure 4). In Ishikawa/ERα cells, E_2_ and all three EDCs induced endogenous *PR* and *GREB* expression. *MCM3* expression was induced only by E_2_ and BPA, whereas *SPUVE* expression was weakly induced by BPA and Zea but not induced by E_2_ or BPAF. Low-dose BPA (1 or 10 nM) antagonizes E_2_-mediated *PR* expression [see Supplemental Material, Figure 3 (http://dx.doi.org/10.1289/ehp.1104689)]. In addition, we confirmed that expression of *PR*, *GREB*, *MCM3*, and *SPUVE* was not induced in Ishikawa/vec cells treated with the three EDCs (data not shown). These data demonstrate that BPA, BPAF, and Zea show direct compound-specific gene regulation in an ER-dependent manner.

## Discussion

*Estrogenic activation of BPA, BPAF, and Zea as agonists or antagonists for ER*α *and ER*β *occurs in a dose/cell type–dependent manner.* In a recent National Health and Nutrition Examination Survey (NHANES), conjugated BPA was detected in the urine of 93% of the U.S. population (Calafat et al. 2009) and this has raised concerns in the medical community (Diamanti-Kandarakis et al. 2009). The present study provides important evidence that the effects of two synthetic EDCs (BPA and BPAF) and one natural EDC (Zea) are hormonally active on ERα and ERβ. Diverse biological effects in a variety of tissues have been attributed to low-dose (≤ 50 ng/mL *in vivo* or ≤ 100 nM *in vitro*) environmental BPA exposure. BPA impedes the activity of endogenous estrogens by disrupting the proper activity of ERs in a diverse set of target tissues (Wetherill et al. 2007). To characterize the full spectrum of possible estrogenic effects of these EDCs, we performed reporter activity assays in cell lines derived from three different tissues; these cell lines have low or no ERα expression and have high transfection efficiency. Estrogenic activation by BPA and BPAF was stronger in all three cell lines when they were co-transfected with ERα. Although the relative binding affinity of BPA for ERα and its capacity to activate ER-dependent transcription is approximately 1,000–10,000 times lower than that of E_2_ or diethylstilbestrol (Kuiper et al. 1998; Lemmen et al. 2004), 100 nM BPA activated ERE-mediated activity for ERα in all three cells lines. In contrast, BPA activated ERβ only in HeLa cells. BPAF, a fluorinated derivative of BPA, is potentially more reactive than its hydroxyphenyl derivative and may be toxic due to the electronegative effects of the CF3 group (Matsushima et al. 2010). In Ishikawa and HepG2 cells, the agonistic effect of BPAF for ERα was stronger than that of BPA (10 nM BPAF vs. 100 nM BPA). Zea and its analogs constitute an important class of endocrine disruptors that do not show acute toxicity but do have estrogenic effects on mammals (Boehme et al. 2009). Parveen et al. (2009) showed similar gene expression profiles in MCF-7 breast cancer cells after 10 nM E_2_ or Zea treatment. In the present study, we used the ERE-luc reporter assay system to verify the estrogenic activation of Zea and found that it has stronger agonistic effect than lower concentrations of E_2_ (1 or 10 nM). However, that activity decreased at higher concentrations (≥ 100 nM) in HepG2 cells (for ERα) and in HeLa cells (for ERβ).

Because of the pleiotropic mechanism of BPA action, BPA is defined as a selective ER modulator that binds to ERs and acts as an agonist in some tissues and as an antagonist in other tissues (Wetherill et al. 2007). Studies have shown that antagonistic effects of BPA at low concentrations inhibit key adipokines, which are proposed to protect humans from inducing metabolic syndrome (Hugo et al. 2008). Using mouse beta TC-6 cells, Makaji et al. (2011) observed that low concentrations of BPA directly affected a decrease in insulin secretion though ERβ. BPAF is a full agonist for ERα but a highly specific antagonist for ERβ in HeLa cells (Matsushima et al. 2010). Gene expression profiling in Ishikawa cells showed that several EDCs, including BPA and Zea, exhibited expression patterns similar to those of ICI, suggesting that these EDCs have mixed estrogenic and antiestrogenic properties (Boehme et al. 2009). To investigate mechanistically the antagonistic effect of BPA and BPAF in the present study, we treated cells with lower doses of these EDCs (1 or 10 nM) in the presence of 10 nM E_2_. We observed significant inhibition of E_2_-mediated ERE reporter activity by BPA for ERα in Ishikawa cells and by BPAF for ERβ in HeLa cells. In contrast, Zea showed only a partial antagonistic effect for ERα in Ishikawa cells. Our results indicate that EDCs, such as BPA, BPAF, or Zea, are not only able to act as ER agonists but also as antagonists, with effects that are dose- and cell-dependent; this supports the results of experimental studies on insulin secretion and obesity (Hugo et al. 2008; Makaji et al. 2011).

*Analysis of BPA, BPAF, and Zea on ER*α *regions of functionality.* Several *in vitro* and *in vivo* study models have been established to better understand the hormonal regulation of E_2_ on classical ERE-mediated function and on tethered-mediated functions of ERα (Burns et al. 2011; Hewitt et al. 2010). The H1 mutant of ERα is incapable of tethered-mediated (AP-1 function) gene activation, and the AA mutant of ERα cannot bind to ERE sequences. In the present study, we observed similar profiles for E_2_ in H1 mutant ERα and WT-ERα with BPA, BPAF, and Zea. No ERE-mediated activation was observed when the AA-ERα mutant was treated with BPA, BPAF, or Zea, thus confirming that ERα activation is predominately via classical ERE-mediated gene responses.

Two acidic activation domains mediate the ligand-dependent transcriptional activity of ERα AF-1 found in the A/B-domain and a hormone-dependent AF-2 located in the ligand-binding domain (Hall and McDonnell 2005; Tzukerman et al. 1994). To examine the ERE-mediated activation of BPA, BPAF, and Zea on AF-1 and AF-2, we used E1-ERα (AF-1 inactive) and AF-2-ERα (AF-2 inactive). BPA, BPAF, and Zea induced ERE-luc reporter activity for E1-ERα, suggesting that activation occurred via the AF-2 function. E_2_-dependent transcriptional activation in AF-2-ERα is reversed by ICI, which activates AF-2-ERα as a full agonist (Arao et al. 2011; Mahfoudi et al. 1995). ERE-mediated reporter activation of AF-2-ERα by Zea was comparable to that of ICI (100 nM), suggesting that Zea acts through both the AF-1 and AF-2 regions. This may explain, at least in part, why activation by Zea is stronger than activation by BPA and BPAF (both of which activate through AF-2 only). Evidence of a recent study (Arao et al. 2011) suggested that tissue-selective AF-1 and AF-2 hormonal response through ERα (Arao et al. 2011); thus the activities of the EDCs may help explain their toxicity in certain tissues.

*Effects of BPA, BPAF, and Zea on rapid action and ER target gene expression.* Rapid action by estrogen or estrogen-like ligands/compounds has been explored in recent years, and many intracellular signaling cascades, such as those involving MAPK/ERK, *src*, or Akt, have been found to play a role in activation in various tissues and cell lines (Hammes and Levin 2007; Madak-Erdogan et al. 2008). In the present study, p44/42 MAPK activation by BPA, BPAF, and Zea was observed only in Ishikawa/ERα cells (not in Ishikawa/vec cells), suggesting that MAPK activation by the EDCs was ERα dependent. In addition, BPA- and BPAF-induced *PR* gene expression was blocked with PP2, an *src* family tyrosine kinase inhibitor, which suggests that the pathways involved in rapid action responses differ among the EDCs.

Our laboratory has reported that similar target genes are induced by BPA and E_2_ in the mouse uterus, as determined by microarray analysis (Hewitt and Korach 2011). In the present study we found that BPA, BPAF, and Zea induced endogenous expression of *PR*, a well-known ER target gene, in Ishikawa cells stably expressing ERα. *GREB*, *MCM3*, and *SPUVE* have been reported to be ER-responsive genes (Henley et al. 2009; Reid et al. 2005). Induction of these target genes by BPA, BPAF, and Zea revealed that gene expression changes are compound specific. In addition, low-dose BPA (1 or 10 nM) antagonized E_2_-mediated expression of *PR*, suggesting that ER target genes may also be involved in the antagonistic effect of BPA on E_2_. Our results suggest that mechanisms involved in activation differ among the EDCs. More importantly, results demonstrate tissue and dose complexities involved in the mechanisms of these EDCs that are relevant to induction of potential tissue selective toxicity.

## Conclusions

BPA and BPAF can function as EDCs by acting as agonists (at higher concentrations; ≥ 10 nM) and as antagonists (at lower concentrations; ≤ 10 nM) for ERα and ERβ. We found these actions to be cell-type specific. BPA and BPAF activated the AF-2 function of ERα, and Zea had strong estrogenic effects due to its ability to activate both AF-1 and AF-2 functions. In addition, BPA, BPAF, and Zea induced the p44/42 MAPK pathway, suggesting that these EDCs can mediate rapid action responses involved in endogenous ER signaling events as well as genomic responses. Taken together, the data demonstrate the mechanistic importance of cell type specificity in evaluation of the potential activities of multiple EDCs.

## Supplemental Material

(434 KB) PDF
