# Supplementary material for: Differential Estrogenic Actions of Endocrine-Disrupting Chemicals Bisphenol A, Bisphenol AF, and Zearalenone through Estrogen Receptor α and β in Vitro
Source: Environ Health Perspect. 2012 Apr 11;120(7):1029–35. doi: 10.1289/ehp.1104689 (PMC3404668; doi:10.1289/ehp.1104689)
Supplement: (434 KB) PDF [file ehp.1104689.s001.pdf]

Supplemental Materials for

Differential Estrogenic Actions of Endocrine-Disrupting Chemicals Bisphenol A, Bisphenol AF and Zearalenone through Estrogen Receptor α and β *in Vitro*

Yin Li, Katherine A. Burns, Yukitomo Arao, Colin J. Luh and Kenneth S. Korach*

Receptor Biology Section, Laboratory of Reproductive and Developmental Toxicology, National Institute of Environmental Health Sciences, National Institutes of Health, Research Triangle Park, North Carolina, USA

Table of Contents

Supplemental Figure 1 -------------------------------------- 2

Supplemental Figure 2 -------------------------------------- 3

Supplemental Figure 3 -------------------------------------- 4

Supplemental Table 1 -------------------------------------- 5

Supplemental Figure 1

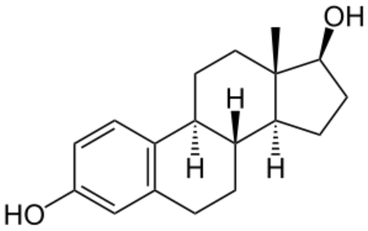

17β-estradiol (E2)

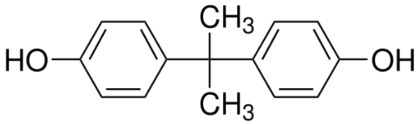

Bisphenol A (BPA)

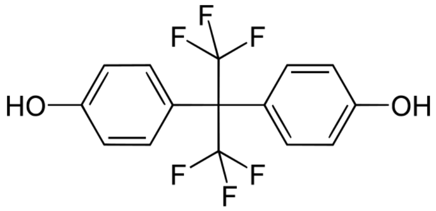

Bisphenol AF (BPAF)

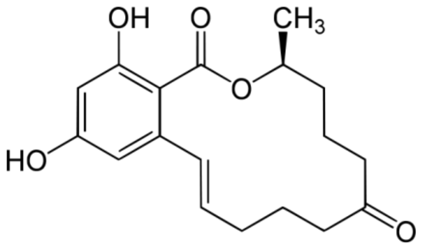

Zearalenone (Zea)

S Figure 1. Chemical structures of E2, BPA, BPAF and Zea

Supplemental Figure 2

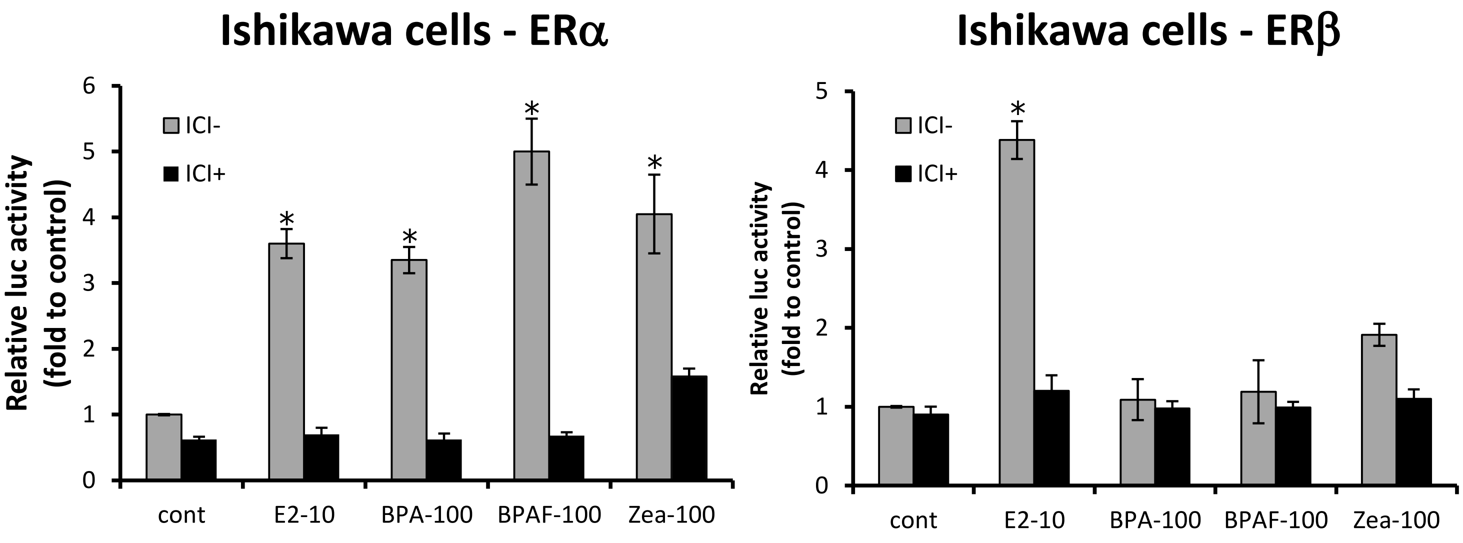

S Figure 2. ERα and ERβ transcriptional activity was blocked by the pure ER antagonist, ICI 182,780 in Ishikawa cells. Cells were transfected with ERE-luc, pRL-TK vector (as inter-control) and pcDNA/WT-ERα or pcDNA/WT-ERβ plasmid overnight. After changing to fresh starve medium, cells were treated with the vehicle (0), 10 nM E2, 100 nM BPA, 100 nM BPAF, 100 nM Zea or treated in combination with 1000 nM ICI for 18 h. The ERE-mediated ERα activation was detected by luciferase reporter assays. Transfection efficiency was normalized by renilla luciferase using pRL-TK. Fold change was calculated relative to the vehicle. Data shown is representative of three independent experiments ± SEM (*, $p < 0.05$ compared with the vehicle).

Supplemental Figure 3

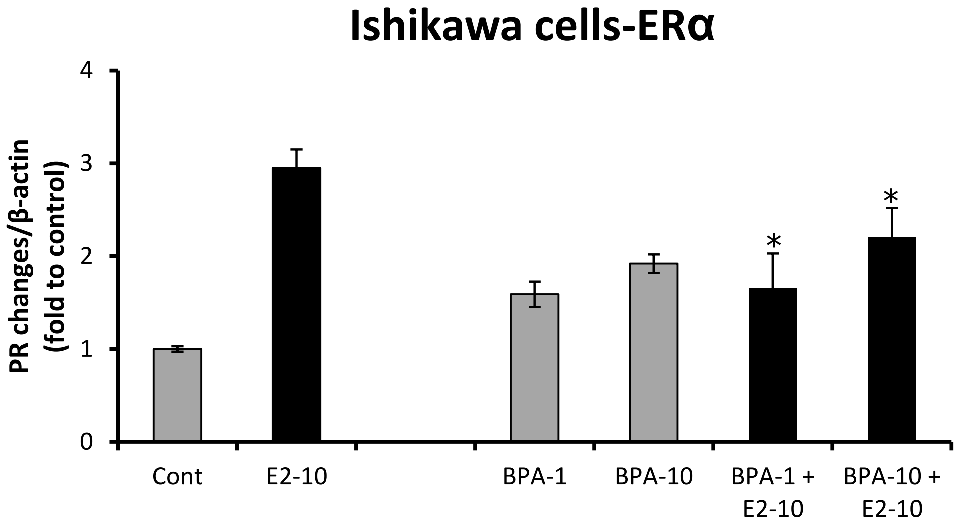

S Figure 3. Low dose of BPA reduced E2-mediated PR gene expression in Ishikawa/ERα cells. Total RNA was extracted from Ishikawa/ERα cells after the vehicle (0), 10 nM E2, 1 or 10 nM BPA, 1 nM BPA and 10 nM E2 or 10 nM BPA and 10 nM E2 treatments for 18 hours. The mRNA levels of PR were quantified by real time-PCR. Experiments were repeated three times and the results are presented as fold change calculated relative to the vehicle (*, $p < 0.05$ compared with 10 nM E2 treatment).

Supplemental Table 1

S Table 1. The primers of real time PCR

Human PR (NM_000926.4):	forward	5'-GACGTGGAGGGCGCATAT-3'
	reverse	5'-GCAGTCCGCTGTCCTTTTCT-3′
Human GREB1 (NM_014668):	forward	5′-CAAAGAATAACCTGTTGGCCC-3′
	reverse	5′-GACATGCCTGCGCTCTCATAC-3′
Human MCM3 (NM_002388):	forward	5′-CTCCTGCTTCCTCAGCTGTGT-3′
	reverse	5′-GGACGACTTTGGGACGAACTA-3′
Human SPUVE (NM_007173):	forward	5′-ATGCCCGAGCAGATGAAATT-3′
	reverse	5′-CCAACCCTTGGGCACATG-3′
Human β-actin (NM_001164317):	forward	5′-GACAGGATGCAGAAGGAGATCAC-3′
	reverse primer	5′-GCTTCATACTCCAGCAGG-3′
